# A systematic and methodological review of attentional biases in eating disorders: Food, body, and perfectionism

**DOI:** 10.1002/brb3.1458

**Published:** 2019-11-07

**Authors:** Christina Ralph‐Nearman, Margaret Achee, Rachel Lapidus, Jennifer L. Stewart, Ruth Filik

**Affiliations:** ^1^ Laureate Institute for Brain Research Tulsa OK USA; ^2^ School of Psychology University of Nottingham Nottingham UK; ^3^ Department of Psychology University of Tulsa Tulsa OK USA; ^4^ Department of Community Medicine University of Tulsa Tulsa OK USA

**Keywords:** anorexia nervosa, attentional bias, binge‐eating disorder, bulimia nervosa, psychophysiology

## Abstract

**Objective:**

The current systematic and methodological review aimed to critically review existing literature utilizing implicit processing, or automatic approach‐ and/or avoidance‐related attentional biases between eating disorder (ED) and nonclinical samples, which (a) highlights how psychophysiological methods advance knowledge of ED implicit bias; (b) explains how findings fit into transdiagnostic versus disorder‐specific ED frameworks; and (c) suggests how research can address perfectionism‐related ED biases.

**Method:**

Three databases were systematically searched to identify studies: PubMed, Scopus, and PsychInfo electronic databases. Peer‐reviewed studies of 18‐ to 39‐year‐olds with both clinical ED and healthy samples assessing visual attentional biases using pictorial and/or linguistic stimuli related to food, body, and/or perfectionism were included.

**Results:**

Forty‐six studies were included. While behavioral results were often similar across ED diagnoses, studies incorporating psychophysiological measures often revealed disease‐specific attentional biases. Specifically, women with bulimia nervosa (BN) tend to approach food and other body types, whereas women with anorexia nervosa (AN) tend to avoid food as well as overweight bodies.

**Conclusions:**

Further integration of psychophysiological and behavioral methods may identify subtle processing variations in ED, which may guide prevention strategies and interventions, and provide important clinical implications. Few implicit bias studies include male participants, investigate binge‐eating disorder, or evaluate perfectionism‐relevant stimuli, despite the fact that perfectionism is implicated in models of ED.

## Graphical abstract

The current systematic and methodological review aimed to critically review existing literature utilizing implicit processing, or automatic approach‐ and/or avoidance‐related attentional biases between eating disorder (ED) and nonclinical samples, which (a) highlights how psychophysiological methods advance knowledge of ED implicit bias; (b) explains how findings fit into transdiagnostic versus disorder‐specific ED frameworks; and (c) suggests how research can address perfectionism‐related ED biases. While behavioral results were often similar across ED diagnoses, studies incorporating psychophysiological measures often revealed disease‐specific attentional biases, suggesting that further integration of psychophysiological and behavioral methods may identify subtle processing variations in ED.

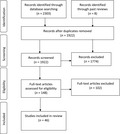

## 1 INTRODUCTION

At least one person's life is taken every 62 min directly from an eating disorder (ED) (Eating Disorders Coalition, 2016), with about a 50% relapse rate within one year of treatment (Keel, Dorer, Franko, Jackson, & Herzog, 2005). Even after over four decades of ED research, key factors underlying the development and maintenance of ED symptomatology and behavior are still unclear. Therefore, it is imperative to clarify biobehavioral mechanisms of ED using multimethod, reliable, valid, and objective metrics to inform prevention and treatment. ED is characterized by abnormal patterns of food consumption in conjunction with heightened emotional intensity, food preoccupation, and/or compensatory behaviors to expel food, such as vomiting, laxatives, or overexercise (Denny, Loth, Eisenberg, & Neumark‐Sztainer, 2013). Three primary EDs prevalent in Western societies over the past 50 years are anorexia nervosa (AN), bulimia nervosa (BN), and binge‐eating disorder (BED) (see Table 1 for DSM‐III through V diagnostic criteria).

**Table 1:** DSM‐III‐R, DSM‐IV, and DSM‐V Diagnostic Criteria for ED

DSM‐III‐R Diagnostic Criteria for ED (APA, 1987)	
AN	Weight loss of 15% expected body weight or failure to make expected weight gain during period of growth
	Intense fear of weight gain, despite underweight
	Disturbance in body image—believes to be fat when underweight
	Amenorrhea (loss of menses or failure to begin menses as expected)
BN	Recurrent episodes of binge eating that include a sense of loss of control
	Recurrent inappropriate compensatory behavior
	Both behaviors occur at least twice/week for a minimum of three months
	Persistent overconcern with shape and weight
BED	Binge eating listed as a feature of bulimia nervosa
DSM‐IV Diagnostic Criteria for ED (APA, 1994)	
AN	Weight loss of 15% expected body weight or failure to make expected weight gain during period of growth
	Intense fear of weight gain, despite underweight
	Disturbance in the way in which one's weight or shape is experienced and/or undue influence of body weight/shape on self‐esteem, and/or denial of seriousness of condition
	Amenorrhea (loss of menses or failure to begin menses as expected)
BN	Recurrent episodes of binge eating that include a sense of loss of control
	Recurrent inappropriate compensatory behavior
	Both behaviors occur at least twice/week for a minimum of three months
	Self‐evaluation unduly influenced by weight or shape
	Does not occur exclusively during episodes of anorexia nervosa
BED	Listed as a descriptor for subsets under "eating disorder not otherwise specified"
DSM‐V Diagnostic Criteria for ED (APA, 2013)	
AN	Intense fear of weight gain, despite underweight
	Disturbance in body image‐ believes to be fat when underweight
	Amenorrhea (loss of menses or failure to begin menses as expected)
BN	Recurrent episodes of binge eating that include a sense of loss of control
	Recurrent inappropriate compensatory behavior
	Both behaviors occur at least twice/week for a minimum of three months
BED	Persistent overconcern with shape and weight
	Binge eating listed as a feature of bulimia nervosa
	Marked distress about binge eating
	Binge eating characterized by ≥3 of: rapid eating; eating until uncomfortably full; eating large amounts when not physically hungry; eating alone because of embarrassment; feeling disgusted, depressed, or guilty after overeating

Abbreviations: AN, anorexia nervosa; BED, binge‐eating disorder; BN, bulimia nervosa.

Although BN was initially recognized as a unique psychological disorder distinct from AN in the DSM‐III (American Psychiatric Association, 1987), key common psychological factors (such as food‐, body‐, and perfectionism‐related cognitions) are theoretically proposed to aid in the development and maintenance of all three EDs (Fairburn, Cooper, & Shafran, 2003). This growing transdiagnostic perspective is based on the high rate of comorbidity and migration between classified EDs; for instance, more than 50% of those with AN move between AN and BN behaviors (Bulik, Sullivan, Fear, & Pickering, 1997; Devlin, Jahraus, & DiMarco, 2010), and many formerly diagnosed with AN or ED not otherwise specified (NOS) are then newly diagnosed with BN or EDNOS (Agras, Walsh, Fairburn, Wilson, & Kraemer, 2000; Fairburn et al., 2003; Sullivan, Bulik, Fear, & Pickering, 1998). Therefore, viewing each ED as a separate psychiatric disorder with distinct underlying mechanisms would lead to many “recovering” from one psychiatric disorder and “developing” a new psychiatric disorder (e.g., transition from AN to BN). Finally, a growing literature has supported the development of the Transdiagnostic Theory for Eating Disorders (Fairburn et al., 2003), in which shared cognitive mechanisms (such as perfectionism‐, body‐, and food‐related mechanisms) are proposed to underlie all three primary EDs. The National Institute of Health's Research Domain Criteria (NIH RDoC) takes the transdiagnostic framework several steps broader, with guidelines to clarify certain cognitive mechanisms which may underlie a variety of psychiatric disorders. For instance, food restraint may be developed or maintained by similar cognitive mechanisms in eating disorders and other common comorbid disorders such as obsessive‐compulsive disorder (see, e.g., Brooks & Schiöth, 2019). The current review aims to specifically evaluate current evidence that support or question three transdiagnostic cognitive mechanisms shared between eating disorders, and not broadly across psychiatric disorders.

Despite the fact that weight‐related core beliefs are thought to automatically influence stimulus processing in ED (Vitousek & Hollon, 1990), many studies solely implement interview or self‐report questionnaires to assess information processing and body image‐related perceptions (see, e.g., Bulik, Sullivan, & Kendler, 2002; Coniglio et al., 2017; Dakanalis et al., 2015; Duarte, Pinto‐Gouveia, & Ferreira, 2014; Gall et al., 2016; Goldschmidt, Lavender, Hipwell, Stepp, & Keenan, 2017; Goldschmidt et al., 2015; Jensen & Steele, 2009; Loth, MacLehose, Bucchianeri, Crow, & Neumark‐Sztainer, 2014; Rohde, Stice, & Marti, 2015; Slane, Burt, & Klump, 2010; Stephen, Rose, Kenney, Rosselli‐Navarra, & Striegel Weissman, 2014; Troisi et al., 2006). While these methods allow for assessment of individuals' conscious belief systems, or “explicit processing", they are unable to access a person's unconscious attentional biases toward or away from specific stimuli, or “implicit processing” that may serve to drive and maintain ED psychopathology. Further development of novel measures may identify subtle processing variations in ED, which may guide prevention strategies and interventions, as well as increase the understanding of information processing strategies involved in the development and maintenance of ED (Smith & Rieger, 2006). The age range 18–39 years old for the current review was preselected to more clearly understand implicit cognitive processing related to eating disorders. That is, to study an age range that includes the most common adult age of onset for an ED (see, e.g., Mangweth‐Matzek & Hoek, 2017), but this age range also avoids the most common onset age range of perimenopause and menopause (see, e.g., Bastian, Smith, & Nanda, 2003). As the decrease in estrogen during the perimenopausal hormonal transition has been reported to alter or decline cognitive processes in a woman's 40s and 50s, as well as produce many physical changes and disturbances, we did not include individuals outside 39 years of age in order to avoid confounding cognitive bias findings related to EDs (see, e.g., Russell, Jones, & Newhouse, 2019; Weber, Maki, & McDermott, 2014).

Prior reviews of attentional biases in clinical ED samples present conflicting patterns of results, suggesting that ED patients show greater attention toward, avoidance of, and/or maintenance of attention to disease‐salient information. This incongruence may be due, in part, to variations in paradigms/stimuli used, as well as conceptualization of EDs as transdiagnostic versus distinct disorders. Prior reviews (see Aspen, Darcy, & Lock, 2013; Brooks, Prince, Stahl, Campbell, & Treasure, 2011; Dobson & Dozois, 2004; Faunce, 2002; Johansson, Ghaderi, & Andersson, 2005; Lee & Shafran, 2004) emphasize food‐ and body‐related biases linked to face‐valid ED symptoms such as eating restriction and altered evaluation of body shape/weight, focusing on behavioral paradigms assessing these biases. The present review updates these behavioral findings and highlights: (a) what psychophysiological measures, as well as assessments that integrate psychological and cognitive states, tell us about implicit processing biases in ED; (b) how implicit bias findings fit into transdiagnostic versus disorder‐specific ED frameworks; and (c) how future research can address the function of perfectionism‐related biases within ED samples. In the sections that follow, peer‐reviewed studies of 18–39‐year‐olds that involve visual attentional biases assessed using pictorial and linguistic stimuli related to food, body, and perfectionism were the focus.

## 2 METHOD

**Figure 1:**
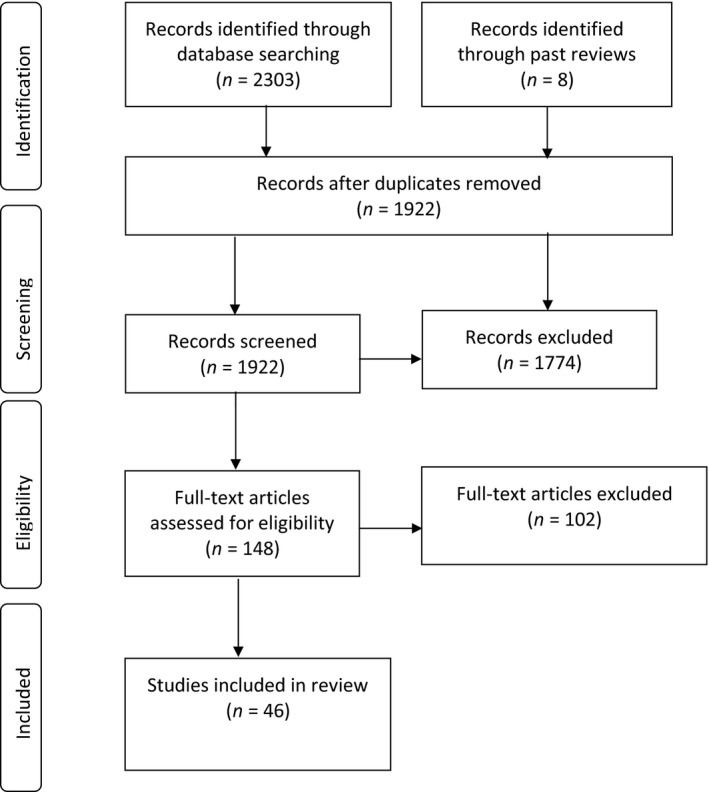
Systematic literature review PRISMA flowchart

The current systematic and methodological review was conducted following PRISMA Guidelines (see Figure 1 PRISMA Flow Chart; Moher, Liberati, Tetzlaff, & Altman, 2009).

### 2.1 Information source, search, eligibility criteria, study selection, and risk of bias in individual studies evaluation

Peer‐reviewed articles written in English were systematically searched in three large electronic databases: PubMed, Scopus, and PsychInfo. No publication date limitations were used. The search was concluded on June 28, 2019. The search terms in the title, abstract, or keywords comprised: (“Body Image Disturbance” OR “Body Dissatisfaction” OR “Body Concerns” OR “Body Image” OR “Food” OR “Perfectionism”) AND (“Eating Disorder” OR “Anorexia” OR “Bulimia” OR “Binge Eating Disorder”) AND (“Attention” OR “Cognitive” OR “Attentional” OR “Information Processing” OR “Dot‐Probe” OR “Neutral” OR “Stroop” OR “Eye‐Tracking” OR “fMRI” OR “EEG” OR “Visual Search Task”). Relevant and applicable studies from past reviews were also obtained. Briefly, after articles and search results from the three databases were manually combined from three independent reviewers in a systematic fashion, and duplicates were removed (*n* = 1,922), remaining titles and abstracts were read thoroughly by two of the independent reviewers to ensure that the following review inclusion and eligibility criteria were met: (a) results based upon original research published in peer‐reviewed journals written in English; (b) human participants aged 18–39 with at least one active AN, BN, and/or BED group according to DSM‐III, DSM‐IV, or DSM‐V criteria who were compared to healthy individuals without ED (HC); and (c) article contained implicit methods to investigate information processing strategies (pictorial or linguistic) involving food‐, body‐ and/or perfectionism‐related stimuli (*n* = 1,774 excluded). Remaining full‐text articles (*n* = 148) were read thoroughly by two independent reviewers, and 46 met full inclusion criteria for this review (Tables 2 and 3 report detailed findings from these studies, whereas Table S1 provides rationale for the exclusion of additional studies). Finally, the 11 applicable criteria items from the Kmet quantitative analysis (Kmet, Lee, & Cook, 2004) were used to assess the risk of bias of each of the remaining 46 studies to evaluate individual studies for risk of bias. Each study was scored 0, 1, or 2 for each of the 11 items (see Table S2a, S2b, and S2c for results) with the maximum score of 22. Any disagreements between reviewers were resolved by consensus between the independent reviewers . Only full texts which met all eligibility criteria were included in the systematic and methodological review.

**Table 2:** Modified Stroop findings in eating disorders (ED) included

Authors	Sample size				Stroop interference			Clinical metrics	
	AN	BN	HC−	HC+	Body	Food	Weight	ED Diagnosis	ED Scales
Ben‐Tovim, Walker, Fok, and Yap (1989)	17T	19T	38	N/A	BN > HC	BN > HC AN > HC	N/A	DSM‐III	N/A
Black et al. (1997)	N/A	16T	13	16	BN = HC included weight	BN = HC	N/A	DSM‐IV	Restraint Questionnaire
Davidson and Wright (2002)	N/A	20T	46	N/A	BN > HC	N/A	BN > HC	DSM‐III‐R	EAT
Fassino et al. (2002)	20A	N/A	20	N/A	AN > HC included weight	AN = HC	N/A	DSM‐IV	EDI−2, BSQ
Green et al. (1999)	34	N/A	39	N/A	AN > HC−	N/A	N/A	DSM‐IV	N/A
Johansson et al. (2008)	13A	20A	31	27	BN > AN BN > HC−	AN > HC−	N/A	DSM‐IV	SED
Jones‐Chesters et al. (1998)	16T	16T	16	N/A	BN > HC AN > HC included weight	BN > HC AN > HC	N/A	DSM‐III‐R	DEBQ‐R, BSQ, EAT
Long et al. (1994)	37T	N/A	45	51 Obese restrictors,	AN > HC− AN = HC+	AN > HC− AN = HC+	N/A	DSM‐III‐R	N/A
Lovell et al. (1997)	31	24	23 AN REC, 11 BN REC, 18 no history	15 no history of ED, current dieters	BN,recovered AN > recovered BN & no ED History includes weight	No interference	N/A	DSM‐III‐R	EDI−2
Perpina et al. (1993)	18T	14T	32	N/A	AN + BN>HC BN > HC	AN + BN>HC AN > HC	N/A	Russell 1970 criteria for AN & Russell 1979 criteria for BN	RS, EDI
Sackville et al. (1998)	20T	N/A	N/A	33 low restraint, 20 high restraint	AN > HC	AN = HC	N/A	DSM‐IV	RS, EDI−2, EAT

Abbreviations: A, in active stage of illness; AN, anorexia nervosa; BN, bulimia nervosa; BSQ, Body Shape Questionnaire; DEBQ‐R, Dutch Eating Behavior Questionnaire; EAT, eating attitudes test; EDE, Eating Disorder Examination; EDI, Eating Disorders Inventory; EDI‐2, Eating Disorders Inventory‐2; HC−, healthy control; HC+, food restrained, driven to thinness, or symptomatic dieters; RS, Restraint Scale; SED, survey for eating disorders; T, in inpatient or outpatient treatment for ED at the time of the study.

**Table 3:** Psychophysiological and non‐Stroop behavioral studies of implicit bias in ED included

Author/Task type	Sample size				Stimuli/Results		Clinical metrics	
	AN	BN	BED	HC	Body	Food	ED Diagnosis	ED scales
fMRI								
Castellini et al. (2013)	21AN‐R, A	N/A	N/A	20 HC−	AN > HC for IFG activation to oversized bodies; AN > HC for middle temporal gyrus activation to undersized bodies; within AN, higher IFG activation to oversized bodies associated with greater ED symptom severity	N/A	DSM‐IV	EDE‐Q
Collins et al. (2017)	N/A	10A	N/A	10	N/A	HC > BN for precuneus, cuneus, and cerebellar activation	DSM−5	EDE
Friederich et al. (2010)	17T	N/A	N/A	18	During self‐other body shape comparisons, AN > HC for insula/supplementary motor cortex activation, but HC > AN for rostral ACC activation. AN > HC anxiety and dissatisfaction with current body shape.	N/A	DSM‐IV	EDE‐Q
Geliebter et al. (2016)	N/A	N/A	10	10	N/A	BED > HC for ACC activation and increased ACC functional connectivity with insula, cerebellum, and supramarginal gyrus to high ED food cues	DSM−5	QEWP, BES, DEBQ
Miyake, Okamoto, Onoda, Kurosaki, et al. (2010)	11T AN‐R; 11T AN‐BP	11T	N/A	11	Self fat‐image: (AN‐R, AN‐BP, HC)> BN for amygdala activation; (AN‐BP, HC)> (AN‐R, BN) for mPFC activation; (AN‐BP, HC)> (AN‐R, BN) for DLPFC activation	N/A	DSM‐IV	EDI−2
Miyake, Okamoto, Onoda, Shirao, et al. (2010)	12T AN‐R; 12T AN‐BP	12T	N/A	12	Negative body image: (AN‐R, AN‐BP)> (BN, HC) for amygdala activation; (AN‐BP, BN)> (AN‐R, HC) for mPFC activation; (AN‐R, AN‐BP)> HC for inferior parietal activation	N/A	DSM‐IV	EDI−2
Mohr et al. (2010)	16	N/A	N/A	16	AN > HC negative rating of actual body; AN > HC body misrepresentation; within AN, insula and mPFC activation to thin self‐images during satisfaction rating task > thin self‐images in the body size estimation task (HC did not show this pattern)	N/A	DSM‐IV	FKB−20
Neveu et al. (2018)	N/A	35T	N/A	26	N/A	BN > HC use of unhealthiness to make food choices; within BN, higher vmPFC activation associated with lower food health ratings (stronger relationship than HC)	DSM IV	EAT 26, EDI 2
Spangler and Allen (2012)	N/A	12T	N/A	12	BN > HC for mPFC activation in fat condition; BN = HC in thin condition	N/A	DSM‐IV	EDDS, EDE
Suchan et al. (2013)	10T	N/A	N/A	15 HC−	Within AN, body‐focused attention associated with middle frontal gyrus, precuneus, cingulate gyrus activation (these regions did not emerge for HC)	N/A	DSM‐IV	Contour Drawing Rating Scale
Suda et al. (2013)	20A	N/A	N/A	15 HC−	Focus on body checking: HC > AN for mPFC and fusiform gyrus activation; within AN, lower medial PFC activation associated with greater body shape concerns	N/A	DSM‐IV	EDE‐Q
Uher et al. (2003)	8A, 9 REC	N/A	N/A	9 HC−	N/A	REC > HC for mPFC, ACC, cerebellum activation, and HC > REC for inferior parietal, occipital activation; REC > AN lateral/apical PFC and ACC activation	DSM‐IV	
Uher et al. (2004)	16T	10T	N/A	19 HC−	N/A	(AN and BN)> HC for OFC, ACC activation; HC > AN for inferior parietal, cerebellum activation; (HC, AN)> BN for lateral PFC activation	DSM‐IV	N/A
Uher et al. (2005)	7 AN‐R, 6 AN‐BP	9T	N/A	18	HC> (AN, BN) for activation in occipital, temporal & inferior parietal regions	N/A	DSM‐IV	EDE‐Q
Van den Eynde et al. (2013)	N/A	21T	N/A	21	BN > HC for insula activation; HC > BN for fusiform gyrus activation	BN = HC	DSM‐IV‐TR	EDE‐Q, FCQ‐T/S, SAAS, PACS
Vocks et al. (2010)	13T	15T	N/A	27	Looking at own body: HC > BN for inferior temporal, inferior parietal, middle frontal gyrus activation; HC > AN for inferior parietal, mPFC, IFG, fusiform, and superior parietal activation. Looking at other's body: AN> (BN, HC) middle/superior temporal activation; AN > HC for insula, precentral, mPFC, fusiform, middle frontal gyrus, inferior parietal, precuneus activation; BN > HC for IFG, middle frontal gyrus, supramarginal gyrus activation; AN > BN for insula, IFG, cingulate, precentral, inferior parietal, supramarginal, precentral activation; AN> (BN, HC) for amygdala activation.	N/A	DSM‐IV‐TR	EDE‐Q, Drive for Thinness, EDI−2, BIAQ
Go/No‐Go								
Brooks et al. (2012)	13 AN‐R, T	N/A	N/A	20	N/A	AN > HC accuracy on N‐back task (working memory) but AN performance compromised; AN < HC accuracy on Go/ No‐Go task (inhibition), but stimuli did not affect AN performance	DSM‐IV	EDI−2
Mobbs et al. (2008)	N/A	18T	18	N/A	BN: Body word RT > neutral word RT; discrimination BN < HC; inhibition BN < HC	BN: Food word RT > neutral word RT; discrimination BN < HC; inhibition BN < HC	DSM‐IV	Drive for thinness, EDI−2, MAC−24
Hemispheric perception								
Kazén et al. (2019)	20T	N/A	N/A	22	AN show distorted body perception when shown images in RVF/LH; distortions reduce with RH activation	N/A	ICD−10‐CM	TCQ‐E
Visual search								
Smeets et al. (2008)	22T	22T	N/A	60	ED < HC, detection time ‐ difference less pronounced for body stimuli compared to neutral	No speeded detection of high‐cal. food; ED > HC distraction by high‐cal foods	DSM‐IV	Restraint Scale, EDE‐Q, EDI−2, BSQ
Anagrams								
Brockmeyer et al. (2018)	40	N/A	N/A	40	AN > HC produced negative sentences. Negative interpretation bias (+) corr w/ ED symptom severity	N/A	DSM−5	EDE‐Q, BSQ
Meyer et al. (2005)	15T AN‐BP; 13T AN‐R	22T	N/A	50	N/A	AN‐BP, AN‐R, BN, & HC process food words more rapidly than neutral or threat; no difference in RT to food words between clinical and nonclinical groups	DSM‐IV	EDI
Word association								
Berry et al. (1998)	19T	21T	N/A	20	N/A	Reaction Time: AN < BN, HC	DSM‐IV	EAT−26
Modified dot‐probe								
Blechert et al. (2010)	19A	18A	N/A	21	AN: saccade latencies for self < other bodies; HC: saccade latencies self = other bodies	N/A	DSM‐IV	EDE‐Q, BIAQ, BCQ
Shafran et al. (2007)	3T	6T	N/A	94	N/A	ED RT. for probe in same location as (‐) & neutral weight < RT for probe in same location as (+) eating stimuli	DSM‐IV	EDE
Eye‐Tracking								
Blechert et al. (2009)	N/A	20	N/A	22	BN longer fixations on bodies/ low BMIs compared to HC; BN show bias toward bodies of low BMI, pattern not replicated in HC & BN own body ratings, (‐) corr. between self‐attractiveness ratings and bias toward low BMI bodies	N/A	DSM‐IV	EDE, BIAQ, BCQ, BISS
George et al. (2011)	16T	N/A	N/A	46	AN > HC distribution of fixations including more upper & lower torso	N/A	DSM‐IV	EDBQ, BSQ
Giel et al. (2011)	14T AN‐R; 5T AN‐BP	N/A	N/A	38	N/A	AN > HC attentional disengagement from food; AN attentional disengagement (+) corr. w/ ED severity	DSM‐IV	EDI−2
Leehr et al. (2018)	N/A	N/A	24A	22+, 26	N/A	BED less adept at disengaging from stimuli & greater difficulty with inhibition	DSM IV	EDE, FCQ‐t, FCQ‐s
Phillipou et al. (2016)	24T&A	N/A	N/A	24	AN hyperscan stimuli (more fixations of short durations) and increased fixations to mid‐heavy male stimuli than female; AN similar saccadic amp. for implicit & explicit tasks, HC different saccadic amp.; AN as accurate as HC in estimate of body size and attended to similar body areas	N/A	DSM−5	EDE‐Q, FRS
Sperling et al. (2017)	N/A	N/A	17	23	N/A	BED > HC attention toward food stimuli; attentional bias in BED marginally negatively associated with BMI and gaze duration negatively associated with BMI & EDE‐Q score	DSM 5	EDE
Von Wietersheim et al. (2012)	35T	N/A	N/A	32	Dwell time on own body AN = HC; Dwell time on thighs AN > HC; fixations on breast area AN < HC; AN (‐) attractive ratings of abdomen corr. with more dwell time	N/A	DSM‐IV‐TR	EDI−2
SEM & EMG								
Friederich et al. (2006)	15T	11T	N/A	30	No startle differences between ED & HC although both ED groups rated “idealized thin” female bodies as highly anxiety provoking	BN < startle response but rated food as highly anxiety provoking. AN = HC startle response	DSM‐IV	N/A
Event‐Related Potentials (ERP)								
Blechert et al. (2011)	22A	22A	N/A	32	N/A	ED enhanced processing of both high and low cal foods while HC higher EPN to high‐cal only	DSM‐IV	EDE‐Q
Positron Emission Tomography (PET)								
Gordon et al. (2001)	8	N/A	N/A	8	N/A	AN > activation in L. occipital cortex and R. temporal/occipital cortex in response to Hi versus Low cal. foods. AN also greater heart rate and feelings of anxiety on exposure to Hi cal. food	DSM‐IV	EAT

Abbreviations: −, negative; +, positive; A, in active stage of illness; ACC, anterior cingulate cortex; Amp, amplitude; AN, anorexia nervosa; AN‐BP, anorexia nervosa binge‐purge subtype; AN‐R, anorexia nervosa restricting subtype; BCQ, Body Checking Questionnaire; BES, Binge Eating Scale; BIAQ, Body Image Avoidance Questionnaire; BISS, Body Image State Scale; BN, bulimia nervosa; BSQ, Body Shape Questionnaire; DEBQ‐R, Dutch Eating Behavior Questionnaire; DLPC, dorsolateral prefrontal cortex; EAT, eating attitudes test; EDDS, Eating Disorder Diagnostic Scale; EDE, Eating Disorder Examination; EDI, Eating Disorders Inventory; EDI‐2, Eating Disorders Inventory‐2; EPN, early posterior negative; FCQ‐s, Food Craving Questionnaire ‐ state; FCQ‐t, Food Craving Questionnaire ‐ trait; FKB‐20, Body Image Questionnaire; FRS, Figure Rating Scale; HC−, healthy control; HC+, food‐restrained, driven to thinness, or symptomatic dieters; L, left; LH, left hemisphere; LPFC, lateral prefrontal cortex; MAC‐24, Anorectic Cognition Questionnaire; mPFC, medial prefrontal cortex; PACS, Physical Appearance Comparison Scale; PFC, prefrontal cortex; QEWP, Questionnaire on Eating & Weight Patterns; R, right; REC AN, recovered anorexia; RH, right hemisphere; RS, Restraint Scale; RT, response time; RVF, right visual field; SAAS, Social Appearance Anxiety Scale; SED, survey for eating disorders; T, in inpatient or outpatient treatment for ED at the time of the study; vmPFC, ventral–medial prefrontal cortex; WM, working memory.

### 2.2 Data collection and synthesis of results

Studies were then grouped by methodology (e.g., Stroop, eye‐tracking, fMRI, etc.), then by eating disorder diagnosis, and then by type of stimuli (i.e., body, food, or perfectionism). Findings include differences between groups for each method, task, and type of stimulus used. A description of each paradigm and/or psychophysiological method used to index implicit attentional bias in ED is provided below, as well as a summary and interpretation of findings across studies employing each approach.

## 3 RESULTS

### 3.1 Behavioral indices of implicit attentional bias

#### 3.1.1 Modified Stroop 

The original Stroop task (1935) requires individuals to voice the color of a color‐related word (red) within varying contexts, showing that participants are faster in voicing a word (red) when the text color is displayed in a congruent color (red) as opposed to an incongruent text color (blue, green, or yellow). This difference in response time is termed Stroop interference, thought to be a measure of implicit processing time to inhibit the word itself, focusing instead on the color in which the word is printed. A modified Stroop paradigm, historically the most common measure for implicit attentional biases in ED (Faunce, 2002), displays an ED‐salient word (fat) in a specific ink color (blue); the participant is then asked to ignore the word meaning and only name the ink color. The longer the color‐naming duration, the greater the interference thought to be related to the word meaning, interpreted by researchers as an indirect measure of attentional bias (Faunce, 2002). ED‐salient words such as “fat” may be threatening or distracting for ED, resulting in greater interference in ED as opposed to other patient or HC groups. As the Stroop interference metric consists of a basic subtraction between response times for ED‐salient and neutral words, this metric alone cannot determine whether the interference is due to hypervigilance (fixating on the word longer and therefore being distracted from the color of the word) or avoidance (avoiding the threatening ED word, and therefore the color of the word).

Across studies reviewed, the majority of Stroop studies support a transdiagnostic theoretical view of ED (Fairburn et al., 2003), demonstrating that active and recovered AN and BN typically show greater Stroop interference than HC to words indexing body shape and/or food, although there are a few reports of disorder‐specific biases (i.e., AN with food or body, and BN with body or weight) (Ben‐Tovim & Morton, 1989; Davidson & Wright, 2002; Fassino et al., 2002; Green, Corr, & Silva, 1999; Johansson, Carlbring, Ghaderi, & Andersson, 2008; Jones‐Chesters, Monsell, & Cooper, 1998; Long, Hinton, & Gillespie, 1994; Lovell, Williams, & Hill, 1997; Perpina, Hemsley, Treasure, & de Silva, 1993; Sackville, Schotte, Touyz, Griffiths, & Beumont, 1998). On the whole, this research supports cognitive theories of ED involving altered processing of food‐ and body‐related stimuli (e.g., Cooper & Fairburn, 1994; Fairburn, 1981; Fairburn, 1984; Fairburn, Shafran, & Cooper, 1999; Williamson, White, York‐Crowe, & Stewart, 2004). Moreover, there is some evidence to support the idea that ED‐related attentional bias cuts across clinical ED and nonclinical groups with particular personality characteristics; for example, HC reporting restrained eating and/or a preoccupation with thinness show a similar interference pattern as AN and BN (Perpina et al., 1993) or a lower interference than AN and BN (Ben‐Tovim & Morton, 1989), and food‐restrained obese individuals show similar Stroop interference as AN (Long et al., 1994).

With respect to methodological issues impacting Stroop interference, two factors appear to influence the degree of attentional bias difference between ED and HC: (a) the use of masked versus unmasked stimuli and (b) the use of blocked versus event‐related stimulus presentation designs. For instance, an innovative Stroop paradigm employed masked, preconscious stimuli (i.e., word covered by five Xs: “XXXXX”) and unmasked, or conscious stimuli to evaluate AN and HC response times with thin and fat body words as well as high‐ and low‐calorie food words (Sackville et al., 1998); although AN and HC show similar responses to masked words, AN exhibit greater interference than HC to unmasked thin, fat, and high‐calorie words, pointing to the importance of conscious processing of word meaning in AN attentional bias. Furthermore, block design presentation (i.e., words presented from one category condition in a set) versus a mixed design (i.e., words presented from a mixture of category conditions in a set) may make the modified Stroop color‐naming task more robust in identifying ED‐specific cognitive interferences to food‐ and body‐related words (Jones‐Chesters et al., 1998); since only one study examines differences between these designs in ED, findings warrant further replication.

Given that Stroop studies vary as a function of ED inclusion/exclusion criteria as well as stimulus content (e.g., food, eating, weight, calories, body size), timing, and presentation duration, it is not surprising that this literature shows some inconsistencies. While much of the research supports biased responding, two studies report no Stroop interference differences between AN and HC for food‐ or eating‐related stimuli (Fassino et al., 2002; Sackville et al., 1998). Moreover, findings are inconsistent regarding whether AN is primarily characterized by attentional bias to body‐based stimuli, or to both body‐ and food‐based stimuli. Finally, although AN and BN appear to show similar patterns when both groups are included within the same study, additional research that does not include AN participants suggests that BN, restrained HC, and unrestrained HC groups do not, in fact, differ in attentional bias to food‐related words (Black, Wilson, Labouvie, & Heffernan, 1997). Discrepancies may also be based on limited statistical power to detect group differences as well as inclusion/exclusion criteria for HC group membership.

#### 3.1.2 Modified dot‐probe

The dot‐probe task (MacLeod, Mathews, & Tata, 1986), unlike the modified Stroop task, can estimate whether an individual's attentional bias reflects hypervigilance (fixed toward) or avoidance (looking away) from a salient or threatening stimulus. In this context, two stimuli (one neutral, such as “chair,” and one threatening, such as “chubby”) are presented together on a computer screen; after they disappear, a probe (typically a black square or fixation cross) appears in the location of one of the two stimuli, and participants are instructed to press a button for the location where they saw the probe (Rieger et al., 1998). If one's attention is initially focused on the ED‐relevant (chubby) stimulus location, researchers expect the participant to respond faster to the probe at that location than at the location of the neutral stimulus (“chair”). One of two dot‐probe studies demonstrates that AN display significantly faster rapid eye movements, or saccades, toward probes presented in locations of self‐body photos compared to other body photos, than HC, whereas BN displayed the opposite pattern (Blechert, Ansorge, & Tuschen‐Caffier, 2010). The findings suggest that BN may engage in more social comparison in their body evaluations than AN, although more research is needed to evaluate this hypothesis. Similarly, the second of the two studies indicates that unspecified clinical ED shows quicker response times to probes indexing locations of food (positive and negative) and body (neutral shape, neutral weight, and negative shape) images than HC (Shafran, Lee, Cooper, Palmer, & Fairburn, 2007). This latter study supports Williamson et al.'s (2004) Integrated Cognitive Behavioral Theory of Eating Disorders, and a transdiagnostic theoretical perspective of ED (Fairburn et al., 2003). While able to differentiate the type of processing strategy and direction (i.e., hypervigilance, maintained attention, difficulty with disengagement, and avoidance) of salient stimuli, the modified dot‐probe task only provides “snapshots” of processing (Starzomska, 2017).

#### 3.1.3 Go/No‐Go

This task requires a participant to make a binary decision on each presented stimulus; next, on the basis of an additional cue, the individual is instructed either to perform a motor response (go), such as pressing a button, or to refrain from making a motor response (no‐go). Go reaction time and no‐go accuracy are typically measures of focused attention and behavioral inhibition, respectively. One study shows that restricting AN exhibit greater errors and longer response times than HC during the presentation of food pictures (high‐/low‐calorie, sweet/savory), which they propose exposes the difficulty for restricting AN to flexibly “set‐shift” between task rules in the presence of subliminal food stimuli (Brooks et al., 2012). Furthermore, this study shows that restricting AN also exhibit greater working memory accuracy than HC, except when subliminal food pictures (overlayed with a mosaic pattern) were presented. The authors suggest that interference during implicit processing of subliminal food is linked to restricting severity in AN. Another study shows that BN exhibit poorer no‐go accuracy than HC, particularly within the context of food‐related stimuli (Mobbs, Van der Linden, d'Acremont, & Perroud, 2008). These findings suggest (a) a similar heightened sensitivity to food pictures as found with self‐body photos found in modified dot‐probe, and (b) that impulsivity associated with binge‐eating supports theories in which food‐related cognitive impairments play a key role in the development and maintenance of BN (Fairburn, 1981, 1984; Williamson et al., 2004).

#### 3.1.4 Visual search

This task measures attention by the speed that an individual is able to visually scan a series of words to detect a target word among several distractors. Quicker detection of disease‐salient targets is thought to index attentional bias toward these stimuli and may therefore indicate the presence of ED symptomatology. One study shows that although restricting AN and purging BN both detect body‐related targets faster than HC, they are slower in detecting food‐related targets (Smeets, Roefs, Van Furth, & Jansen, 2008); these findings suggest two transdiagnostic biases in ED, one toward bodily evaluation, and the other away from food items, which are likely construed as threatening.

#### 3.1.5 Anagrams

A unique study assessed restricting subtype AN, binge‐purge subtype AN, BN, and HC performance on an anagram‐solving task that included food‐related, ego‐threat, and neutral words (Meyer et al., 2005). Since all groups solved food words (e.g., eack = cake) more quickly than ego‐threat words (e.g., lfia = fail), this task design failed to index ED‐sensitive attentional bias. A similar paradigm, the scrambled sentences task (SST; Wenzlaff & Bates, 1998), requires participants to unscramble sentences that can then be rearranged into both a grammatically correct positive and negative sentence. In contrast to Meyer et al. (2005), this paradigm demonstrated that AN created more sentences depicting a negative interpretation of their bodies than HC (Brockmeyer et al., 2018), consistent with the idea that bodily aversion is a core pathology contributing to AN.

#### 3.1.6 Word association

Within the context of a word association test assessing perceptual hypersensitivity to various groups of words, AN exhibit slower response times specifically to food‐related words than HC, who in turn did not differ from BN (Berry, Kelly, Canetti, & Bachar, 1998). These results support theories advocating for a food‐based attentional bias as a key component of AN.

### 3.2 Psychophysiological indices of implicit attentional bias

#### 3.2.1 Eye‐tracking

Eye‐tracking is a noninvasive technique enabling researchers to measure eye movement, direction of eye gaze, and duration of eye approach (fixation) or avoidance of various stimuli. Since eye‐tracking can provide continuous metrics of information processing in the order of milliseconds, it is a remarkable tool that can be employed to directly investigate visually based attentional biases (Liversedge & Findlay, 2000). Four studies comparing eye‐tracking in AN demonstrate that, compared to HC, they: (a) overestimate body size, fixating on wider regions of the body (including the groin area up to the collarbone, focusing on the bony areas of the torso, such as the hipbone/collarbone) when judging attractiveness of images depicting various body mass indices (BMI) (George, Cornelissen, Hancock, Kiviniemi, & Tovée, 2011); (b) rate body images with lower BMI as more attractive (George et al., 2011); (c) spend less time fixating on their own breasts and more time fixating on their own thighs, judging themselves as less attractive (von Wietersheim et al., 2012); (d) engage in a greater number of short fixations upon body‐related stimuli, suggestive of increased anxiety, despite no fixation differences between groups on specific regions (Phillipou et al., 2016); and (e) fixate less on food‐related pictures at late but not early stages of stimulus processing, a pattern consistent with active avoidance of threat‐related cues (Giel et al., 2011). Similar to AN, BN fixate longer than HC on comparison bodies with lower BMIs than higher BMIs. Since this gaze pattern is positively related to greater body dissatisfaction post‐task, findings support the assertion that BN is characterized by upward bodily comparisons (Blechert, Nickert, Caffier, & Tuschen‐Caffier, 2009). Finally, eye‐tracking studies of BED indicate that they (a) have more difficulty inhibiting saccadic eye movements to food‐related images after a negative mood induction than both overweight and normal‐weight HC groups, a pattern linked to heightened impulsivity (Leehr et al., 2018) and (b) can identify food‐related target images in a visual search task faster than other types of targets when compared to HC (Sperling, Baldofski, Lüthold, & Hilbert, 2017). Taken together, eye‐tracking findings point to food avoidance in AN, social comparison in BN, and food‐related approach behavior in BED, results that support more disorder‐specific ED mechanisms as opposed to transdiagnostic markers of ED as a unitary construct.

#### 3.2.2 Startle eye‐blink modulation (SEM) and facial electromyography (EMG)

Electromyography uses small receptors placed along certain muscular areas of the face to measure the amplitude of an eye‐blink reflex that is larger than baseline, as well as startle amplitude inhibition (i.e., eye‐blink reflex that is smaller than baseline) that is elicited during the startle eye‐blink modulation (SEM) paradigm. During SEM, the individual is often presented with a variety of visual stimuli with and without a startle‐eliciting stimulus, such as a loud, startling noise. SEM is thought to effectively index attentional and emotional processing to ED‐salient stimuli (Filion, Dawson, & Schell, 1998). When encountering food‐ and body‐related images along with a startling auditory noise, one study indicates that BN show a smaller startle eye‐blink response (thought to reflect less aversion) to food pictures than AN and HC (Friederich et al., 2006); although no group differences in startle emerged to body images, results are consistent with BN having an approach‐related food bias, as opposed to AN, who tend to have an avoidance‐related bias to food cues.

#### 3.2.3 Event‐related potentials (ERP)

Electroencephalography **(**EEG) is used to measure the brain's neural activity recorded from the scalp in response to various events, and ERPs consist of EEG responses to a particular event averaged over many trials. ERPs are typically quantified by the peak amplitude and latency of a particular response in milliseconds, and ERP responses occurring within the first 300 milliseconds poststimulus are thought to reflect stimulus perception, discrimination, and attention processes. AN and BN both exhibit larger ERP amplitudes within this time window (thought to reflect greater neural resources devoted) to both low‐ and high‐calorie food images than neutral images (Blechert, Feige, Joos, Zeeck, & Tuschen‐Caffier, 2011), findings consistent with an early food‐based attentional bias in AN and BN, in line with a transdiagnostic perspective.

#### 3.2.4 Positron emission tomography (PET)

Positron emission tomography detects changes in blood flow and therefore indirectly measures the brain's neural activity to various stimuli (as opposed to EEG, which directly measures neuronal signals). During PET scanning, a radioactive drug (called a “tracer”) is injected into the bloodstream of participants in order to aid detection of blood flow to specific areas of the brain. One such PET study demonstrates that in response to high‐ but not low‐calorie food images, AN exhibit greater heart rate, anxiety ratings, and blood flow to temporal and occipital brain regions than HC (Gordon et al., 2001). Since these regions are involved in perceptual and attentional processing of visual stimuli, these results support the notion of a bias associated with high‐calorie food, as opposed to food more generally, as reported by ERP study findings (Blechert et al., 2011). However, since ERPs are measured in the order of milliseconds, whereas PET imaging reveals blood flow changes in the order of seconds, it may be the case that AN are indeed initially drawn to food‐based stimuli more generally, but then experience narrows their bias to high‐calorie foods.

#### 3.2.5 Functional magnetic resonance imaging (fMRI)

Functional magnetic resonance imaging is a method that, like PET, indirectly measures neuronal activity by measuring blood flow and oxygenation changes over time. Due to its high spatial resolution, fMRI studies can clarify which brain areas and networks are involved with specific mental processes in the temporal order of seconds. Unlike most ED Stroop studies, which investigate attentional biases to both body‐ and food‐related words within the same sample, fMRI studies (*n* = 16) rarely incorporate both but evaluate one or the other, most often employing images instead of words.

For instance, fMRI studies focusing on responses to body‐related images suggest that AN, when compared to HC, show: (a) divergent brain signal patterns to self‐referential images (Mohr et al., 2010; Suda et al., 2013) and other human bodies (Friederich et al., 2010; Suchan et al., 2013; Suda et al., 2013); (b) lower attentional resources (e.g., insula and/or prefrontal cortex, or PFC) devoted to processing their own image as opposed to ideal underweight images (Castellini et al., 2013; Mohr et al., 2010); and (c) less effective connectivity of the visual system network (including the occipital cortex), which is argued to alter body‐size processing and contribute to body‐size overestimation (Mohr et al., 2010; Suchan et al., 2013). Similarly, another fMRI study investigating responses to food images demonstrates that underweight AN exhibit lower PFC activation to food than recovered AN and HC, who did not differ from each other (Uher et al., 2003). Similar to AN findings for body pictures, results suggest heightened visual attention paid to disorder‐salient stimuli (food) unless one is in an actively undernourished state. Given that PFC activation is often associated with cognitive control processes, elevated PFC signal to ideal body and food pictures could be related to issues regarding control over diet and thinness. Finally, an additional study does not compare fMRI patterns of brain activation in AN and HC, but instead evaluates contributions of left versus right hemispheric activation to visual processing, presenting images of the participant's body in either the left or right visual field (Kazén, Baumann, Twenhöfel, & Kuhl, 2019). Prior to viewing these images, participants are primed with positive, negative, or neutral words and then are asked to estimate whether the image is thinner than, equal to, or “fatter” than their own body. Findings indicate that in contrast to HC, AN exhibit perceptual body distortions only after being primed with negative words and viewing pictures presented to their right visual field (corresponding to left hemisphere processing) (Kazén et al., 2019). It may be the case that left hemisphere attentional processing involving cognitive control is ramped up during aversive body‐related thoughts in AN.

A growing literature is investigating brain processes in BN compared to HC alone or in conjunction with AN subtypes. For example, compared to HC, BN exhibit (a) higher medial PFC activation to overweight body images (Spangler & Allen, 2012); (b) lower anterior cingulate cortex (ACC), occipital, and orbitofrontal activation to desirable food cues following a stress induction (Collins et al., 2017); and (c) lower health appraisal of food despite similar ventromedial PFC activation and tastiness appraisals when making desirable food choices (Neveu et al., 2018). Taken together, these findings are consistent with heightened brain resources devoted to social comparison in BN as well as lowered food‐related conflict in the face of stress. In addition, to address potential transdiagnostic conceptualizations of ED, six fMRI studies employed a divergent set of tasks to compare brain responses between AN, BN, and HC; four focusing on body and two focusing on food. First, restricting AN, binge‐purging AN and HC exhibit greater amygdala activation to self‐overweight images (i.e., images of their own body that had been morphed into a larger/overweight body than their actual body size) than BN (Miyake, Okamoto, Onoda, Kurosaki, et al., 2010), and both types of AN display greater amygdala responses to negatively valenced body words than BN (Miyake, Okamoto, Onoda, Shirao, et al., 2010); these findings suggest that not all EDs are characterized by heightened aversive responses to large body shapes. Second, AN exhibit greater amygdala responses to other‐female body images (as opposed to self‐body images) than BN and HC (Vocks et al., 2010), again showing discrepancies in aversion as a function of ED type. In contrast, two fMRI studies report that AN and BN process body‐related information similarly, with both groups showing: (a) lower parietal/occipital (fusiform) responses than HC during viewing of body shapes (Uher et al., 2005) and (b) greater medial PFC responses to negatively valenced body words than HC (Miyake, Okamoto, Onoda, Shirao, et al., 2010).

In response to food pictures, results point toward support for transdiagnostic approaches to ED. For instance, AN and BN rate food images as more aversive than HC, showing greater orbitofrontal and ACC activation to food pictures compared to HC, paired with lower PFC, parietal cortex, and cerebellar activation to these images (Uher et al., 2004). This study suggests that heightened brain resources are being devoted to food valuation and conflict processing across ED groups. Furthermore, the sole fMRI study investigating both body‐ and food‐related stimuli reports that BN display greater insula activation than HC when evaluating themselves against images of slim women, potentially reflecting greater bodily self‐focus (Van den Eynde et al., 2013).

When compared to AN and BN, brain mechanisms involved in BED are relatively understudied. The sole fMRI study of this disorder demonstrates that BED show greater activation in ACC to high‐ than low‐calorie food images when compared to obese HC and healthy weight HC groups, suggestive of heightened resources needed to maintain cognitive control in the face of disorder‐salient stimuli that trigger lack of control in BED (Geliebter, Benson, Pantazatos, Hirsch, & Carnell, 2016).

## 4 DISCUSSION

The current systematic and methodological review employing PRISMA Guidelines (Moher et al., 2009) evaluates patterns of food‐, body‐, and/or perfectionism‐related implicit visual information processing strategies in three types of ED (AN, BN, and BED), comparing them to HC (*n* = 46). Whether various paradigms and psychophysiological approaches supported disorder‐specific versus transdiagnostic substrates of ED was determined. First, although the majority of Stroop studies show that AN and BN both exhibit greater interference to body and shape words than HC, consistent with a transdiagnostic view of ED (Fairburn et al., 2003), Stroop interference calculations are unable to disentangle whether longer response times to these words reflect approach versus avoidance processes (e.g., preoccupation with word meaning vs. looking away from the word entirely). Fortunately, brain imaging, dot‐probe, and eye‐tracking studies, among others, provide more clues as to whether approach versus avoidance mechanisms are involved in these attentional biases. Taken together, findings from these studies suggest that AN are (a) preoccupied with their own as opposed to others' body‐related stimuli when given the choice (Blechert et al., 2010), unless these stimuli are specifically associated with thinness or their ideal underweight body type (Castellini et al., 2013; Mohr et al., 2010; Van den Eynde et al., 2013); (b) show aversion and anxiety toward their own bodies (Brockmeyer et al., 2018; Kazén et al., 2019; Miyake, Okamoto, Onoda, Kurosaki, et al., 2010; Miyake, Okamoto, Onoda, Shirao, et al., 2010) as well as other overweight bodies (Vocks et al., 2010); and (c) are initially drawn to food stimuli (Blechert et al., 2011; Brooks et al., 2012; Gordon et al., 2001) but may switch to avoidance of food, particularly if the food is high‐calorie (Berry et al., 1998; Giel et al., 2011). On the whole, research suggests that BN: (a) focus substantial attention on others' bodies (Blechert et al., 2010, 2011, 2009; Smeets et al., 2008; Spangler & Allen, 2012; Van den Eynde et al., 2013); (b) appear to be more drawn to than repelled by food stimuli (Berry et al., 1998; Blechert et al., 2011; Brooks et al., 2012; Friederich et al., 2006, Smeets et al., 2008; but see Uher et al., 2004); and (c) show difficulty inhibiting behavior in the presence of food cues (Mobbs et al., 2008) as well as reduced conflict‐related brain signals to desirable foods when stressed (Collins et al., 2017). Finally, available work suggests that BED (a) show heightened conflict‐related brain signals to high‐calorie food (Geliebter et al., 2016) similar to AN and BN (Uher et al., 2004); (b) show greater impulsivity and difficulty focusing attention on various stimuli not limited to body and food (Leehr et al., 2018); and (c) may have an approach‐related food bias (Sperling et al., 2017).

As brain imaging paradigms varied widely in task design as well as particular types and/or subtypes of ED, additional research is warranted to replicate these findings. A few studies show that AN and BN show similar patterns of brain activation to body shapes, food pictures, and negatively valenced body words (Miyake, Okamoto, Onoda, Shirao, et al., 2010; Uher et al., 2004, 2005), findings consistent with a transdiagnostic conceptualization of ED. Heightened amygdala responses in AN (Miyake, Okamoto, Onoda, Shirao, et al., 2010; Vocks et al., 2010) may be related to aversion to stimuli that signal weight gain, whereas heightened insula responses in AN and BN (Van den Eynde et al., 2013) may be related to abnormal body image perception. Brain regions involved in cognitive control of behavior (PFC) and detection of conflict/errors (ACC) also appear to be disrupted as a function of ED, with AN showing heightened PFC activation to stimuli signifying thinness (Castellini et al., 2013; Mohr et al., 2010), but AN, BN, and BED all exhibiting heightened ACC activation to food (Geliebter et al., 2016; Uher et al., 2004). These findings are consistent with the need for body control in AN and the issue of food as a primary obstacle to weight control across EDs. Moreover, multiple studies demonstrate altered visual processing in AN (Blechert et al., 2011; Brooks et al., 2012; Gordon et al., 2001; Mohr et al., 2010; Sachdev, Mondraty, Wen, & Gulliford, 2008; Uher et al., 2005) and BN (Blechert et al., 2011; Collins et al., 2017; Uher et al., 2005) to body and/or food stimuli, although more information is warranted to determine the timing of this particular impairment with respect to other potential brain impairments (e.g., insula, amygdala, ACC, and PFC alterations). Like the Stroop task, fMRI methodology is still not ideal to investigate the visual nature of attentional biases; while fMRI may help elucidate the brain activity associated with certain types of stimuli, it cannot clarify the areas of the stimuli that are fixated upon or avoided, for how long, or what regions of interest are included in the visual information processing style. Therefore, it may not be clear what aspects of processing are linked to which areas, and different brain areas may be active in a variety of processes. Many studies support a cognitive theoretical understanding that body‐ and/or food‐related information processing style is an emotion‐activating process that underlies ED, as the presence of stress or negatively valenced stimuli appears to exacerbate differences between ED and HC (e.g., Collins et al., 2017; Kazén et al., 2019; Miyake, Okamoto, Onoda, Shirao, et al., 2010).

Although not widely utilized thus far in ED research, eye‐tracking methodology and EEG/ERP methods appear well suited to evaluate temporal information processing strategies in relation to disease‐salient stimuli to investigate underlying mechanisms of ED, as well as specific tracking of stimulus attention in space. Eye‐tracking is particularly advantageous in that: (a) it allows for collection of continuous measures of multiple types of eye movements in the order of milliseconds, unlike fMRI, which possesses a temporal resolution in the order of seconds; (b) it requires fewer trials to robustly investigate processing strategies than fMRI and EEG/ERP methods, thereby reducing participant fatigue; and (c) it does not exclude people who cannot be exposed to the magnetic nature of fMRI recording. It would be ideal if research evaluating implicit biases in ED integrate one or more psychophysiological methods (e.g., eye‐tracking and ERPs or fMRI) with behavioral performance to decipher whether particular biases reflect approach versus avoidance of various stimuli. This paradigm may be expanded to incorporate both visual and linguistic materials and to include perfectionism‐related stimuli, along with body‐ and food‐related stimuli, as there were no studies investigating implicit perfectionism‐related attentional biases, which may be a key underlying mechanism of ED.

While perfectionism is included in many theoretical models as both a risk factor and underlying cognitive mechanism which perpetuates ED (Fairburn, Cooper, Doll, & Welch, 1999; Lilenfeld et al., 1998, 2000; Schmidt & Treasure, 2006; Slade & Dewey, 1986), this review highlights that there are no studies directly investigating attentional biases or implicit cognitive processing of perfectionism‐related stimuli. This is surprising given that over two decades have passed since Vitousek and Orimoto (1993) suggested the importance of understanding and investigating attentional biases pertaining to perfectionism‐related information. Furthermore, many theories point to perfectionism‐, along with some body‐ and/or food‐related mechanisms, as key to ED, such as: (a) the Two‐Factor Vulnerability‐Stress Model (Joiner, Heatherton, Rudd, & Schmidt, 1997); (b) the Three‐Factor Interactive Model (Vohs, Bardone, Joiner, Abramson, & Heatherton, 1999); (c) the Cognitive‐Interpersonal Model of Anorexia Nervosa (Schmidt & Treasure, 2006; Treasure & Schmidt, 2013); and (d) the Transdiagnostic Model of Eating Disorders (Fairburn et al., 2003). Also, perfectionism is suggested to be a key factor in the etiology of ED in prior studies and reviews (Bardone‐Cone, Sturm, Lawson, Robinson, & Smith, 2010; Egan, Wade, & Shafran, 2011; Jacobi, Hayward, Zwaan, Kraemer, & Agras, 2004; Lilenfeld, Wonderlich, Riso, Crosby, & Mitchell, 2006; Stice, 2002). Therefore, perfectionism‐related information processing style is a significant factor that needs objective investigation in order to clarify its role in attentional biases related to ED tendencies in a similar way as food‐ and body‐related processing are studied.

### 4.1 Comparison with prior reviews

Unlike previous attentional bias reviews including body‐ and/or food‐related stimuli (e.g., Brooks, Prince, et al., 2011; Dobson & Dozois, 2004; Faunce, 2002; Jiang & Vartanian, 2018; Johansson et al., 2005; Kerr‐Gaffney, Harrison, & Tchanturia, 2019; Lee & Shafran, 2004), the current review reveals that fMRI was the primary psychophysiological measure used to measure implicit bias in ED, surpassing purely behavioral modified Stroop paradigms that were previously the focus. Historically, fMRI has been more commonly used in anxiety attentional bias studies, finding that fear‐related information processing translates to heightened amygdala activation and suggests an automatic hypervigilance toward threat‐relevant stimuli (Cisler & Koster, 2010). In the current review, heightened amygdala responses in AN may suggest emotional attentional bias to ED‐threatening words and pictures (either hypervigilance toward or avoidance from stimuli). Thus, a top‐down cognitive control mechanism (e.g., PFC) may be responsible for regulating attentional bias to ED‐salient stimuli that may be interpreted as threatening.

### 4.2 Limitations

Most of the studies used the modified Stroop color‐naming task to investigate the visual attentional biases and information processing strategies of eating disorder‐salient stimuli, which may be an indirect measure, as researchers are unable to know the direction of attentional biases (toward or away from stimuli). Multiple types of stimuli were also used across studies, some of which merged food‐ and body‐related information into one category, rendering it impossible to elucidate the individual contribution of each attentional bias. Others separated food and body stimuli, but there were differences such as using general, positive, and/or negative stimuli (e.g., a whole meal vs. “fattening” or “dieting” foods). Some studies differentiated eating disorder diagnoses (primarily investigating participants with AN), while other studies took a transdiagnostic approach, making it difficult to discern whether attentional bias differs across disorders. Most studies only included females, and therefore, the study of information processing tendencies in males with EDs is missing. Responses related to perfectionism‐related stimuli are also missing in the literature, which may be a key underlying factor in the development and maintenance of ED symptomatology (e.g., Fairburn et al., 2003).

## CONFLICT OF INTEREST

All authors have stated no conflicts of interest.
