# Supplementary material for: A systematic and methodological review of attentional biases in eating disorders: Food, body, and perfectionism
Source: Brain Behav. 2019 Nov 7;9(12):e01458. doi: 10.1002/brb3.1458 (PMC6908865; doi:10.1002/brb3.1458)
Supplement: Supplementary file 1 [file BRB3-9-e01458-s001.docx]

**Supplemental Material**

**Table S1.** List of Excluded Papers

| Aviram-Friedman et al. (2018) | Age of Participants |
| --- | --- |
| Bauer et al. (2017) | Age of Participants |
| Ben-Tovim & Walker (1991) | Age of Participants |
| Brooks et al. (2011a) | Age of Participants |
| Dondzilo et al. (2018) | Age of Participants |
| Gagnon et al. (2018) | Age of Participants |
| Green et al. (1998) | Age of Participants |
| Jonker et al. (2019) | Age of Participants |
| Lane et al. (2017) | Age of Participants |
| Mobbs et al. (2011) | Age of Participants |
| Neimeijer et al. (2017) | Age of Participants |
| Sachdev et al. (2008) | Age of Participants |
| Sackville et al. (1997) | Age of Participants |
| Stormark & Torkildsen (2004) | Age of Participants |
| Redgrave et al. (2008) | Age of Participants |
| Urgesi et al. (2014) | Age of Participants |
| Channon et al. (1988) | Age of Participants not reported |
| Cooper et al. (1992) | Age range of Participants not reported |
| Cooper & Fairburn (1992) | Age of Participants not reported |
| Cooper & Todd (1997) | Age of Participants not reported |
| Ellison et al. (1998) | Age of Participants not reported |
| Green et al. (1994) | Age of Participants not reported |
| Baldofski et al. (2017) | No active ED group and/or no control group |
| Bohon & Stice (2011) | No active ED group and/or no control group |
| Boon et al. (2000) | No active ED group and/or no control group |
| Boutelle et al. (2016) | No active ED group and/or no control group |
| Brooks et al. (2011a) | No active ED group and/or no control group |
| Carter et al. (2000) | No active ED group and/or no control group |
| Cooper & Fairburn (1994) | No active ED group and/or no control group |
| Cowdrey et al. (2011) | No active ED group and/or no control group |
| Devrim et al. (2018) | No active ED group and/or no control group |
| Flynn & McNally (1999) | No active ED group and/or no control group |
| Formea & Burns (1996) | No active ED group and/or no control group |
| Franzen et al. (1988) | No active ED group and/or no control group |
| Hege et al. (2015) | No active ED group and/or no control group |
| Jansen et al. (2005) | No active ED group and/or no control group |
| Kodama et al. (2018) | No active ED group and/or no control group |
| Kostopoulou et al. (2013) | No active ED group and/or no control group |
| Legenbauer et al. (2011) | No active ED group and/or no control group |
| Matheson et al. (2018) | No active ED group and/or no control group |
| Mauler et al. (2006) | No active ED group and/or no control group |
| Mazzurega et al. (2018) | No active ED group and/or no control group |
| Placanica et al. (2002) | No active ED group and/or no control group |
| Ralph-Nearman & Filik (2018) | No active ED group and/or no control group |
| Rieger et al. (1998) | No active ED group and/or no control group |
| Rieger et al. (2017) | No active ED group and/or no control group |
| Riva et al. (1999) | No active ED group and/or no control group |
| Schmitz et al. (2014) | No active ED group and/or no control group |
| Schmitz et al. (2015) | No active ED group and/or no control group |
| Sturman et al. (2017) | No active ED group and/or no control group |
| Svaldi et al. (2010) | No active ED group and/or no control group |
| Tammela et al. (2010) | No active ED group and/or no control group |
| Trentowska et al. (2014) | No active ED group and/or no control group |
| Van Ens et al. (2019) | No active ED group and/or no control group |
| Vocks et al. (2011) | No active ED group and/or no control group |
| Wallis et al. (2018) | No active ED group and/or no control group |
| Wonderlich et al. (2017) | No active ED group and/or no control group |
| Brockmeyer et al. (2019) | Intervention |
| Bulik et al. (1998) | Intervention |
| Keizer et al. (2018) | Intervention |
| Hildebrandt et al. (2018) | No Implicit Visual Attentional Bias |
| Holsen et al. (2012) | No Implicit Visual Attentional Bias |
| Legenbauer et al. (2017) | No Implicit Visual Attentional Bias |
| Legenbauer et al. (2018) | No Implicit Visual Attentional Bias |
| Léonard et al. (1998) | No Implicit Visual Attentional Bias |
| Mergen et al. (2018) | No Implicit Visual Attentional Bias |
| Milos et al. (2017) | No Implicit Visual Attentional Bias |
| Molbert et al. (2018) | No Implicit Visual Attentional Bias |
| Neveu et al. (2014) | No Implicit Visual Attentional Bias |
| Probst et al. (1998) | No Implicit Visual Attentional Bias |
| Steinglass et al. (2015) | No Implicit Visual Attentional Bias |
| Torres et al. (2011) | No Implicit Visual Attentional Bias |
| Trentowska et al. (2017) | No Implicit Visual Attentional Bias |
| Vocks et al. (2007) | No Implicit Visual Attentional Bias |
| Waller & Hodgson (1996) | No Implicit Visual Attentional Bias |
| Biezonski et al. (2016) | Resting-state structural brain study |
| Bulik et al. (1996) | Biological or pharmacological study |
| Bulik et al. (1998) | Biological or pharmacological study |
| Cambridge et al. (2013) | Biological or pharmacological study |
| Chamberlain et al. (2012) | Biological or pharmacological study |
| Holsen et al. (2014) | Biological or pharmacological study |
| Kim et al. (2014) | Biological or pharmacological study |
| Dickson et al. (2008) | Memory/recall with no implicit information processing style measurement |
| Hermans et al. (1998) | Memory/recall with no implicit information processing style measurement |
| Blume et al. (2019) | Review article |
| Brooks et al. (2011b) | Review article |
| Challinor et al. (2017) | Review article |
| De Carvalho et al. (2017) | Review article |
| Esposito et al. (2016) | Review article |
| Kakoschke et al. (2018) | Review article |
| Kerr-Gaffney et al. (2018) | Review article |
| Kober et al. (2018) | Review article |
| Murnen & Smolak (2019) | Review article |
| Stojek et al. (2018) | Review article |
| Lloyd et al. (2018) | Review article |
| Cardi et al. (2015) | Non-visual stimuli |
| Eshkevari et al. (2014) | Non-visual stimuli |
| Monje Moreno et al. (2014) | Non-visual stimuli |
| Oberndorfer et al. (2013) | Non-visual stimuli |
| Setsu et al. (2017) | Non-visual stimuli |
| Wagner et al. (2015) | Non-visual stimuli |
| Mendlewicz et al. (2001) | Unable to obtain full text of manuscript |

Table.S2.a. Kmet quantitative analysis (Kmet, Lee, & Cook, 2004) YES (2), PARTIAL (1), NO (0), N/A

| Criteria | QUESTION | Ben-Tovim et al. 1989 | Black et al. 1997 | Davidson & Wright 2002 | Fassino et al. 2002 | Green et al. 1999 | Johansson et al. 2008 | Jones-Chesters et al. 1998 | Long et al. 1994 | Lovell et al. 1997 | Perpina et al. 1993 | Sackville et al. 1998 | Castellini et al., 2013 | Collins et al., 2017 | Friederich et al., 2010 | Geliebter et al. 2016 | Miyake et al., 2010a |
| --- | --- | --- | --- | --- | --- | --- | --- | --- | --- | --- | --- | --- | --- | --- | --- | --- | --- |
| 1 | Question/objective sufficiently described? | 2 | 2 | 2 | 2 | 2 | 2 | 2 | 2 | 2 | 2 | 2 | 2 | 2 | 2 | 2 | 2 |
| 2 | Study design evident and appropriate? | 2 | 2 | 2 | 2 | 2 | 2 | 2 | 2 | 2 | 2 | 2 | 2 | 2 | 2 | 2 | 2 |
| 3 | Method of subject/comparison group selection or source of information/input variables described and appropriate? | 1 | 2 | 2 | 2 | 2 | 2 | 2 | 2 | 2 | 2 | 2 | 2 | 2 | 2 | 2 | 2 |
| 4 | Subject (and comparison group, if applicable) characteristics sufficiently described? | 1 | 1 | 2 | 2 | 2 | 2 | 2 | 2 | 2 | 2 | 2 | 2 | 2 | 2 | 2 | 2 |
| 5 | If interventional and random allocation was possible, was it described? | N/A | N/A | N/A | N/A | N/A | N/A | N/A | N/A | N/A | N/A | N/A | N/A | N/A | N/A | N/A | N/A |
| 6 | If interventional and blinding of investigators was possible, was it reported? | N/A | N/A | N/A | N/A | N/A | N/A | N/A | N/A | N/A | N/A | N/A | N/A | N/A | N/A | N/A | N/A |
| 7 | If interventional and blinding of subjects was possible, was it reported? | N/A | N/A | N/A | N/A | N/A | N/A | N/A | N/A | N/A | N/A | N/A | N/A | N/A | N/A | N/A | N/A |
| 8 | Outcome and (if applicable) exposure measure(s) well defined and robust to measurement/misclassification bias? Means of assessment reported? | 2 | 2 | 2 | 2 | 2 | 2 | 2 | 2 | 2 | 2 | 2 | 2 | 2 | 2 | 2 | 2 |
| 9 | Sample size appropriate? | 1 | 1 | 2 | 1 | 2 | 1 | 1 | 2 | 2 | 1 | 1 | 1 | 1 | 2 | 1 | 2 |
| 10 | Analytic methods described/justified and appropriate? | 2 | 2 | 2 | 2 | 2 | 2 | 2 | 2 | 2 | 2 | 2 | 2 | 2 | 2 | 2 | 2 |
| 11 | Some estimate of variance is reported for the main results? | 2 | 2 | 2 | 2 | 2 | 2 | 2 | 2 | 2 | 2 | 2 | 2 | 2 | 2 | 2 | 2 |
| 12 | Controlled for confounding? | 1 | 2 | 2 | 2 | 2 | 2 | 2 | 2 | 2 | 2 | 2 | 2 | 2 | 2 | 2 | 2 |
| 13 | Results reported in sufficient detail? | 2 | 2 | 2 | 2 | 2 | 2 | 2 | 2 | 2 | 2 | 2 | 2 | 2 | 2 | 2 | 2 |
| 14 | Conclusions supported by the results? | 2 | 2 | 2 | 2 | 2 | 2 | 2 | 2 | 2 | 2 | 2 | 2 | 2 | 2 | 2 | 2 |
|  | Total Score | 18 | 20 | 22 | 21 | 22 | 21 | 21 | 22 | 22 | 21 | 21 | 21 | 21 | 22 | 21 | 22 |

Table.S2.b. Example of Kmet quantitative analysis (Kmet, Lee, & Cook, 2004) YES (2), PARTIAL (1), NO (0), N/A

| Criteria | QUESTION | Miyake et al., 2010b | Mohr et al., 2010 | Neveu et al., 2018 | Spangler & Allen, 2012 | Suchan et al., 2013 | Suda et al., 2013 | Uher et al., 2003 | Uher et al., 2004 | Uher et al., 2005 | Van den Eynde et al., 2013 | Vocks et al., 2010 | Brooks et al. 2012 | Mobbs et al. 2008 | Kazen et al 2019 | Smeets et al. 2008 | Brockmeyer et al. 2018 |
| --- | --- | --- | --- | --- | --- | --- | --- | --- | --- | --- | --- | --- | --- | --- | --- | --- | --- |
| 1 | Question/objective sufficiently described? | 2 | 2 | 2 | 2 | 2 | 2 | 2 | 2 | 2 | 2 | 2 | 2 | 2 | 2 | 2 | 2 |
| 2 | Study design evident and appropriate? | 2 | 2 | 2 | 2 | 2 | 2 | 2 | 2 | 2 | 2 | 2 | 2 | 2 | 2 | 2 | 2 |
| 3 | Method of subject/comparison group selection or source of information/input variables described and appropriate? | 2 | 2 | 2 | 2 | 2 | 2 | 2 | 2 | 2 | 2 | 2 | 2 | 2 | 2 | 2 | 2 |
| 4 | Subject (and comparison group, if applicable) characteristics sufficiently described? | 2 | 2 | 2 | 2 | 2 | 2 | 2 | 2 | 2 | 2 | 2 | 2 | 2 | 2 | 2 | 2 |
| 5 | If interventional and random allocation was possible, was it described? | N/A | N/A | N/A | N/A | N/A | N/A | N/A | N/A | N/A | N/A | N/A | N/A | N/A | N/A | N/A | N/A |
| 6 | If interventional and blinding of investigators was possible, was it reported? | N/A | N/A | N/A | N/A | N/A | N/A | N/A | N/A | N/A | N/A | N/A | N/A | N/A | N/A | N/A | N/A |
| 7 | If interventional and blinding of subjects was possible, was it reported? | N/A | N/A | N/A | N/A | N/A | N/A | N/A | N/A | N/A | N/A | N/A | N/A | N/A | N/A | N/A | N/A |
| 8 | Outcome and (if applicable) exposure measure(s) well defined and robust to 2measurement/misclassification bias? Means of assessment reported? | 2 | 2 | 2 | 2 | 2 | 2 | 2 | 2 | 2 | 2 | 2 | 2 | 2 | 2 | 2 | 2 |
| 9 | Sample size appropriate? | 2 | 2 | 2 | 2 | 1 | 2 | 1 | 2 | 1 | 2 | 1 | 2 | 2 | 2 | 2 | 2 |
| 10 | Analytic methods described/justified and appropriate? | 2 | 2 | 2 | 2 | 2 | 2 | 2 | 2 | 2 | 2 | 2 | 2 | 2 | 2 | 2 | 2 |
| 11 | Some estimate of variance is reported for the main results? | 2 | 2 | 2 | 2 | 2 | 2 | 2 | 2 | 2 | 2 | 2 | 2 | 2 | 2 | 2 | 2 |
| 12 | Controlled for confounding? | 2 | 2 | 2 | 2 | 2 | 2 | 2 | 2 | 2 | 2 | 2 | 2 | 2 | 2 | 2 | 2 |
| 13 | Results reported in sufficient detail? | 2 | 2 | 2 | 2 | 2 | 2 | 2 | 2 | 2 | 2 | 2 | 2 | 2 | 2 | 2 | 2 |
| 14 | Conclusions supported by the results? | 2 | 2 | 2 | 2 | 2 | 2 | 2 | 2 | 2 | 2 | 2 | 2 | 2 | 2 | 2 | 2 |
|  | Total Score | 22 | 22 | 22 | 22 | 21 | 22 | 21 | 22 | 21 | 22 | 21 | 22 | 22 | 22 | 22 | 22 |

Table.S2.c. Example of Kmet quantitative analysis (Kmet, Lee, & Cook, 2004) YES (2), PARTIAL (1), NO (0), N/A

| Criteria | QUESTION | Meyer et al. 2005 | Berry et al. 1998 | Blechert et al. 2010 | Shafran et al. 2007 | Blechert et al. 2009 | George et al. 2011 | Giel et al. 2011 | Leehr et al., 2018 | Phillipou et al. 2016 | Sperling et al., 2017 | Von Wietersheim et al 2012 | Friederich et al. 2006 | Gordon et al. 2001 |
| --- | --- | --- | --- | --- | --- | --- | --- | --- | --- | --- | --- | --- | --- | --- |
| 1 | Question/objective sufficiently described? | 2 | 2 | 2 | 2 | 2 | 2 | 2 | 2 | 2 | 2 | 2 | 2 | 2 |
| 2 | Study design evident and appropriate? | 2 | 2 | 2 | 2 | 2 | 2 | 2 | 2 | 2 | 2 | 2 | 2 | 2 |
| 3 | Method of subject/comparison group selection or source of information/input variables described and appropriate? | 2 | 2 | 2 | 2 | 1 | 1 | 2 | 2 | 2 | 2 | 2 | 2 | 2 |
| 4 | Subject (and comparison group, if applicable) characteristics sufficiently described? | 2 | 2 | 2 | 2 | 2 | 2 | 2 | 2 | 2 | 2 | 2 | 2 | 2 |
| 5 | If interventional and random allocation was possible, was it described? | N/A | N/A | N/A | N/A | N/A | N/A | N/A | N/A | N/A | N/A | N/A | N/A | N/A |
| 6 | If interventional and blinding of investigators was possible, was it reported? | N/A | N/A | N/A | N/A | N/A | N/A | N/A | N/A | N/A | N/A | N/A | N/A | N/A |
| 7 | If interventional and blinding of subjects was possible, was it reported? | N/A | N/A | N/A | N/A | N/A | N/A | N/A | N/A | N/A | N/A | N/A | N/A | N/A |
| 8 | Outcome and (if applicable) exposure measure(s) well defined and robust to measurement/misclassification bias? Means of assessment reported? | 2 | 2 | 2 | 2 | 2 | 2 | 2 | 2 | 2 | 2 | 2 | 2 | 2 |
| 9 | Sample size appropriate? | 2 | 2 | 1 | 2 | 2 | 1 | 1 | 2 | 2 | 2 | 2 | 1 | 1 |
| 10 | Analytic methods described/justified and appropriate? | 2 | 2 | 2 | 2 | 2 | 2 | 2 | 2 | 2 | 2 | 2 | 2 | 2 |
| 11 | Some estimate of variance is reported for the main results? | 2 | 2 | 2 | 2 | 2 | 2 | 2 | 2 | 2 | 2 | 2 | 2 | 2 |
| 12 | Controlled for confounding? | 2 | 1 | 2 | 2 | 1 | 2 | 2 | 2 | 2 | 1 | 1 | 1 | 2 |
| 13 | Results reported in sufficient detail? | 2 | 2 | 2 | 2 | 2 | 2 | 2 | 2 | 2 | 2 | 2 | 2 | 2 |
| 14 | Conclusions supported by the results? | 2 | 2 | 2 | 2 | 2 | 2 | 2 | 2 | 2 | 2 | 2 | 2 | 2 |
|  | Total Score | 22 | 21 | 21 | 22 | 20 | 20 | 21 | 22 | 22 | 21 | 21 | 20 | 21 |
